# GINSA: an accumulator for paired locality and next-generation small ribosomal subunit sequence data

**DOI:** 10.1093/bioinformatics/btae152

**Published:** 2024-03-19

**Authors:** Eric Odle, Samuel Kahng, Siratee Riewluang, Kyoko Kurihara, Kevin C Wakeman

**Affiliations:** Department of Natural History Sciences, Graduate School of Science, Hokkaido University, Sapporo, Hokkaido 060-0810, Japan; Department of Oceanography, University of Hawaii at Manoa, Honolulu, HI 96822, United States; Institute for the Advancement of Higher Education, Hokkaido University, Sapporo, Hokkaido 060-0817, Japan; Department of Natural History Sciences, Graduate School of Science, Hokkaido University, Sapporo, Hokkaido 060-0810, Japan; Department of Natural History Sciences, Graduate School of Science, Hokkaido University, Sapporo, Hokkaido 060-0810, Japan; Institute for the Advancement of Higher Education, Hokkaido University, Sapporo, Hokkaido 060-0817, Japan; Graduate School of Science, Hokkaido University, Sapporo, Hokkaido 060-0810, Japan

## Abstract

**Motivation:**

Motivated by the challenges of decentralized genetic data spread across multiple international organizations, GINSA leverages the Global Biodiversity Information Facility infrastructure to automatically retrieve and link small ribosomal subunit sequences with locality information.

**Results:**

Testing on taxa from major organism groups demonstrates broad applicability across taxonomic levels and dataset sizes.

**Availability and implementation:**

GINSA is a freely accessible Python program under the MIT License and can be installed from PyPI via pip.

## 1 Introduction

Advances in nucleic acid sequencing technologies have led to a rapid increase in the amount of available genetic data (Keen *et al.* 1996, Warburton and Sebra 2023). To better organize and share this emergent abundance of sequence data between researchers, public databases such as GenBank (Benson *et al.* 1993, Strasser 2011), the European Nucleotide Archive (ENA) (Burgin *et al.* 2023), and the DNA Data Bank of Japan (DDBJ) (Tanizawa *et al.* 2023) were established in the 1980s. For example, evolutionary biologists often rely on small ribosomal subunit rRNA gene (SSU) sequences archived in these databases to study new species. However, sequence databases do not require a complete set of metadata (e.g. site of collection, date of collection, species-level identification, or link to publication) when uploading sequences. Absence of a complete set of metadata can lead to the omission of locality data, forcing biologists to manually seek associated location information elsewhere. To address this disconnect among archived data, we developed GINSA (GbIf Next-gen Sequence Accumulator): a biodiversity research tool that fetches SSU sequences and their associated localities. This tool takes advantage of the Global Biodiversity Information Facility (GBIF) [www.gbif.org], which links taxa (scientific names), localities (sites of occurrence), and SSU sequences.

Manually pairing high-volume SSU sequence and locality data is prohibitively slow. Although GBIF provides links to specific sequences used for identification, researchers must currently follow a convoluted chain of websites to FASTA files stored in off-site repositories (typically ENA for next-generation sequencing). Upon downloading FASTA/FASTQ files, researchers must then manually search massive lists (often hundreds of thousands) of SSU sequences. This time-consuming step is required for each species occurrence on GBIF, for which there can be thousands. Finally, researchers must manually trace sequences back to their occurrence locality from GBIF. We developed GINSA to automate this process.

Previous attempts to address the inaccessibility of sequence metadata include pysrabd (Choudhary 2019), grabseqs (Taylor *et al.* 2020), and *ffq* (Gálvez-Merchán *et al.* 2023). While helpful for specific applications, these tools address use cases that differ from those of GINSA. Python package pysradb provides convenient access to next-generation sequences stored on the National Center for Biotechnology Information (NCBI) Sequence Read Archive but does not focus on data from ENA. Another tool, grabseqs, automates next-generation sequence acquisition for multiple repositories, but requires users to have prior knowledge of the specific accession numbers associated with their organism of interest. Similarly, *ffq* addresses the difficulty in acquiring sequence metadata from ENA. However, *ffq* requires database accession or article DOI numbers as input. In contrast, GINSA leverages the structure provided by GBIF to link specific taxa with their known localities and SSU sequences. Users simply enter the name of their target organism (taxon), and then wait for the collection process to finish automatically.

## 2 Applications and implementation

The GINSA tool provides researchers with large datasets in line with the Big Data (De Mauro *et al.* 2016, Miralles *et al.* 2020) nature of current molecular taxonomy. For instance, evolutionary biologists rely on large molecular sequence datasets to study speciation trends (Schlegel 1991, Adl *et al.* 2019). Large datasets help resolve cryptic diversity, which is a challenge seen across the tree of life—animals (Marchán *et al.* 2018, Li and Wiens 2023), plants (Vieu *et al.* 2023, Windham *et al.* 2023), fungi (Koufopanou *et al.* 1997, Pringle *et al.* 2005), bacteria (Meyer *et al.* 2023), protists (Wakeman and Leander 2013, Krienitz *et al.* 2015, Martin *et al.* 2016), archaea (Câmara *et al.* 2023), and viruses (Roux *et al.* 2019). Specialists across a range of taxa can therefore use GINSA to collect more data for their phylogenetic (SSU sequence) and biogeographic (locality) analyses.

There are currently over 2.6 billion occurrences on GBIF representing 1.3 million confirmed species. Occurrence coverage is uneven across taxa; animals account for 79.8%, followed by plants at 16.8%, and other taxa at <1.5% each (Table 1). When considering only next-generation SSU sequences archived by ENA (via the publisher MGnify), coverage favors bacteria and protists (Supplementary Fig. S1). This ENA/MGnify subset includes approximately 23.7 million GBIF occurrences, which together comprise the pool of data accessible by GINSA. Moreover, this data pool is expected to grow. Since 2008, 25–50 thousand new species from each major global region have been added to GBIF every two years (Waller 2020).

**Table 1.** Summary of GBIF biodiversity coverage across major groups.^a^

Taxon	Total occurrences	ENA/MGnify
Animals	2 097 448 406	33 065
Plants	442 531 533	376 547
Fungi	38 914 204	955 943
Bacteria	22 722 639	18 355 383
Protists	15 895 213	3 098 014
Archaea	442 031	335 722
Viruses	910 025	0
Incertae sedis	8 014 894	630 700

a Coverage on GBIF for each major group is quantified by number of occurrences. Column 1 (Taxon) lists the major groups of life recognized by GBIF. Groups Chromista and Protozoa are combined as Protists. Column 2 (Total Occurrences) shows the current total number of GBIF occurrences. Column 3 (ENA/MGnify) shows the number of occurrences based on material samples archived by MGnify.

**Figure 1.**
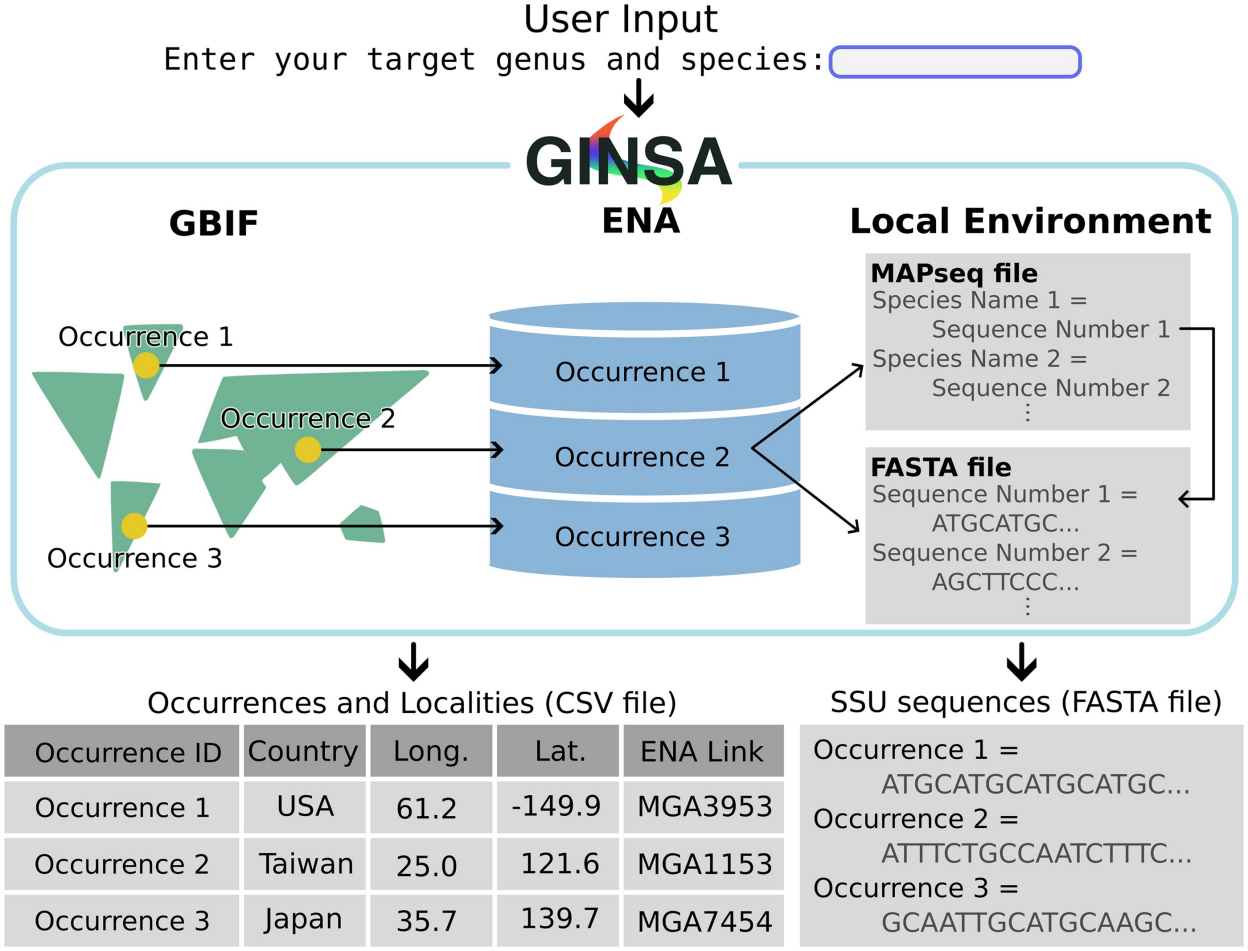
Chart visualizing the GINSA workflow. User input is taken as a GBIF search taxon. Occurrences are then linked with their source sequences archived on ENA. Output CSV and FASTA files link GBIF occurrence IDs, localities, and sequences.

The GINSA tool offers an efficient method for accessing ENA next-generation sequence repositories linked to GBIF taxon occurrences (Fig. 1). Users enter a search taxon, then GINSA queries GBIF for all recorded occurrences of that taxon. Next, the program extracts respective ENA links from GBIF occurrence records, downloading and processing FASTA/FASTQ files into a curated list of SSU sequences. The following details outline how GINSA automates this task.

### 2.1 User prompt

Upon running GINSA, users are prompted for two inputs: the project folder path and the target taxon. All subsequent sub-folders and output files are saved inside the project folder. Search taxa are parsed in Python as a string, and users may enter either one-word (e.g. genus name *Lecudina*) or two-word (e.g. species name *Lecudina longissima*) queries.

### 2.2 GBIF taxon search

An API call searches GBIF for all instances of the queried taxon, and a list is generated by the function search_species_occurrences() for all matching GBIF occurrences.

### 2.3 FASTA and MAPseq download

Sequential API calls are made to ENA/MGnify for each occurrence linked from GBIF. Next, the function ssu_fasta_grab() downloads FASTA/FASTQ files containing SSU sequences belonging to the search taxon. This process is then repeated by mapseq_grab() for the associated MAPseq files. These MAPseq files are necessary because next-generation sequencing read assembly generates long lists of sequences with complex names.

### 2.4 SSU contig decode

For each occurrence, a text search is run within the MAPseq file to locate all sequences associated with the search taxon. Next, sequence labels are tracked to specific sequences in the corresponding FASTA/FASTQ file.

### 2.5 Generate FASTA master file

Extracted SSU sequences are gathered into a file named seq_master.FASTA alongside a corresponding metadata table named occurrences.csv. Users may annotate seq_master.FASTA with additional GBIF metadata (e.g. latitude, longitude, or country of origin). A script (misc/suffix_annotator.py) demonstrating annotation with occurrences.csv is provided on the project GitHub repository.

## 3 Availability and testing

The GINSA project was written using Python (Van Rossum and Drake 1995) version 3.12 and is free to use under the MIT License. Code was structured into two scripts: a command line interface (CLI) implementation named GINSA_cli.py and a graphical user interface (GUI) implementation named GINSA_gui.py. Following installation via pip,

pip3 install GINSA

the CLI can be run with a single line of text:

GINSA_cli <path/to/project/directory> <"search taxon">

Moreover, the GUI can be run by simply entering:

GINSA_gui

A broad range of taxonomic groups were represented when testing GINSA (Table 2). These groups include animals (arthropod genus *Lambia*), plants (*Aneura mirabilis*, *Chrysymenia brownii*), fungi (*Malassezia globosa*), bacteria (*Altibacter lentus*), protists (*Lecudina longissima*, *Tetraselmis marina*, *Lecudina tuzetae*, and *Labyrinthula* spp.), and archaea (*Nanohaloarchaea*). This set of taxa allowed us to evaluate the speed and utility of GINSA across multiple taxa and occurrence sizes. Testing was performed on an Intel Xeon W-2235 CPU 3.80 GHz system with 31.0 GiB of available memory running Linux kernel 5.15.0–87. Network download speed during testing was stable, ranging from 443 to 540 Mbp.

**Table 2.** Summary of test taxa occurrences, process runtimes, and output directory sizes.^a^

Taxon	OC (*n*)	RT (min)	Size (GB)
*Lambia* spp.	11	3.4	0.8
*Lecudina longissima*	26	10.1	6.80
*Tetraselmis marina*	190	36.0	5.26
*Nanohaloarchaea*	253	152.2	58.3
*Lecudina tuzetae*	309	86.8	31.9
*Aneura mirabilis*	549	65.3	0.118
*Altibacter lentus*	628	336.0	79.3
*Chrysymenia brownii*	655	95.1	2.21
*Malassezia globosa*	1379	327.5	97.8
*Labyrinthula* spp.	2602	593.0	178.5

a Occurrences (OC) reflect the number (n) of Global Biodiversity Information Facility (GBIF) entries corresponding to a particular search taxon. Processing runtimes (RT) are measured in minutes (min). Resulting output directory sizes (Size) are measured in gigabytes (GB).

Following testing, GINSA exhibited applicability across a spectrum of taxa and occurrence sizes. Taxa with smaller datasets (*Lambia* spp., *Lecudina longissima*) took less time to analyze than taxa with larger datasets (*Malassezia globosa*, *Labyrinthula* spp.) (Table 2). All tests completed without interruption, although the larger taxa required significantly more storage (97.8–178.5 GB). Network speed and local storage capacity were the only observed bottlenecks to performance. With sufficient storage and internet connectivity, taxa with an even greater number of GBIF occurrences could theoretically be analyzed using GINSA.

Neither runtime nor output directory size were linearly associated with occurrence count. These observations are attributed to the presence of GBIF occurrences identified through means (e.g. human identification, museum specimens, and Sanger sequencing) other than next-generation sequencing. For example, although *Aneura mirabilis* had 549 occurrences on GBIF, only two of those occurrences linked back to next-generation SSU sequences. For this reason, GINSA generates an output plot summarizing the proportion of searched occurrences containing next-generation SSU sequence data. Examples of these plots are provided on the project GitHub page (https://github.com/ericodle/GINSA).

## 4 Conclusion

This article introduced GINSA (GbIf Next-gen Sequence Accumulator), a novel tool designed to bridge the gap between genetic sequence data and locality metadata. Rapid growth in the amount of data from next-generation sequencing technologies has generated increasing demand for more efficient methods to pair sequence information with biogeographic context. The GINSA tool addresses this challenge by automating the collection of SSU sequences and locality metadata for a given taxon through integration with the Global Biodiversity Information Facility (GBIF). By streamlining the process of accessing and pairing these crucial data, GINSA enables researchers to work more efficiently. This tool has a beginner-friendly design, open-source code base, and is applicable across major organism groups. As such, GINSA is offered as a free resource for evolutionary biologists navigating the complexities of cryptic speciation and Big Data research.

## Supplementary Material

btae152_Supplementary_Data

## Data Availability

All code and associated data for GINSA are freely available on the project repository at https://github.com/ericodle/GINSA.
